# Radiological and Thermal Dose Correlations in Pallidothalamic Tractotomy With MRgFUS

**DOI:** 10.3389/fsurg.2019.00028

**Published:** 2019-05-17

**Authors:** Marc N. Gallay, David Moser, Christian Federau, Daniel Jeanmonod

**Affiliations:** ^1^SoniModul, Center for Ultrasound Functional Neurosurgery, Solothurn, Switzerland; ^2^ETH Zurich, Institute for Biomedical Engineering, University Zurich, Zurich, Switzerland; ^3^Department of Radiology, University Hospital Basel, Basel, Switzerland

**Keywords:** MRgFUS (magnetic resonance-guided focused ultrasound surgery), Parkinson's disease, functional neurosurgery, pallidothalamic tractotomy, incisionless, thermal dose, imaging, radiology

## Abstract

**Background:** MR-guided focused ultrasound (MRgFUS) offers the possibility of safe and accurate lesioning inside the brain. Until now, most MRgFUS thermal applications have been based on temperature or energy protocols. Experimental studies support however an approach centered on thermal dose control.

**Objective:** To show the technical feasibility and lesion size predictability of a thermal dose approach during MRgFUS pallidothalamic tractotomy (PTT) against chronic therapy-resistant Parkinson's disease (PD).

**Methods:** MR and thermal dose data were analyzed in 31 MRgFUS interventions between January and December 2017 in patients suffering from chronic therapy-resistant Parkinson's disease (PD) using a standardized PTT target covered by 5 to 7 target lesion sub-units.

**Results:** Good correlations were found between (1) the mean axial T2 lesion diameter intraoperatively and the mean 240 cumulative equivalent min at 43°C (240 CEM) thermal dose diameter (*r* = 0.52), (2) the mean axial T2 diameter 48 h post-treatment and the mean 18 CEM thermal dose diameter (*r* = 0.62), and (3) the mean axial T2 diameter intraoperatively and 48 h post-treatment (*r* = 0.62).

**Conclusion:** Our current approach using a thermal dose steering for multiple target lesion sub-units could be reproduced in 31 interventions with a good lesion size predictability.

## Introduction

Our previous experience of lesioning the pallidothalamic tract on its way to the thalamus in PD (1) has led us, after analysis of recurrences or partial symptom control, to develop a targeting protocol aiming at improved spatial lesion coverage of the target. Our current approach, served by a histological reappraisal of the pallidothalamic tract, adopts the strategy of applying a set of small thermolesions with shortest possible sonication durations, under thermal dose control and moving the focal point of the MRgFUS system onto preplanned coordinates (2). This is a study of thermal dose and imaging correlations aiming to show the technical feasibility and lesion size predictability applying a thermal dose approach, and to compare it with currently used approaches primarily based on temperature or energy protocols. The thermal dose approach is supported by experimental data on focused ultrasound based thermal tissue lesioning (3–5), which predict a 100% lesion probability at the value of 240 CEM.

## Methods

Our current atlas-based spatial coverage technique of MRgFUS PTT described in a companion paper (2) was applied in 31 interventions in 29 chronic therapy-resistant Parkinson's patients. Mean age at treatment was 67 ± 11 years. The targeting accuracy of our MRgFUS intervention experience has been published between 2012 and 2018 (1, 6–9). Clinical results will be presented elsewhere.

Focused ultrasound procedure were performed using the ExAblate Neuro device (InSightec, Haifa, Israel) in a 3-Tesla MR imaging system (GE Discovery 750, GE Healthcare, Milwaukee, WI, USA).

We applied a standardized PTT target lesioning by moving the focal point of the focused ultrasound system onto 5 to 7 preplanned positions. Each sonication had the shortest possible duration and the corresponding power in order to provide a thermal dose of 240 CEM at each focal point. This represents a conservative value corresponding to a 100% probability of lesion (3–5) in a volume of 1.5 × 1.5 × 3.0 mm corresponding to the focal region as given by the manufacturer. According to McDannold (3, 4), an 18 CEM thermal dose represents a 50% probability for thermal damage. The ExAblate software provides integration and graphic 2D-representation of the applied thermal doses of 240 and 18 CEM for each performed sonication. Diameters of thermal dose surfaces were measured on axial MR planes directly on the ExAblate software. No volumetric thermal dose assessment was performed.

To obtain 240 CEM in each target sub-unit, some sonications had to be repeated. Because it is not established yet in which way the thermal dose of repetitive sonications on the same spot may be cumulated, the goal was always to reach a thermal dose of 240 CEM in one sonication.

Intraoperative MR T2 examinations were performed at the end of the intervention as early as possible after the last sonication, with the body coil and with the patient in treatment position. Postoperative MR examinations with the 32-channel head coil were performed 2 days after the intervention, except in one patient, who received it 1 day after. Diameters of thermal lesions were measured on axial T2 MR planes. No volumetric lesion assessment was performed. The thermal lesions, consisting of zones I and II according to Martin et al. (10) were clearly distinguishable from perilesional edema.

Three additional cases were treated before this period and serve to demonstrate the necessity and applicability of the strategy presented here.

Corticosteroids were routinely applied in all patients treated with this protocol, intravenously within 1 h following last sonication (usually 40 mg Solumedrol) and after 12 h. Dexamethasone (3 × 2 mg per day) was given p.o. the following 3–4 days.

Correlations were assessed with Pearson's coefficient with agreement considered good for 0.4 < r ≤ 0.75, and excellent for 0.75 < r.

All patients treated with this protocol signed an informed consent form after having been fully informed about the treatment, its results and risks. No ethical approval was sought because MRgFUS PTT is approved by the Swiss Health State Department, is the subject of a state registry and is covered by swiss social insurances.

## Results

**Figure 1:**
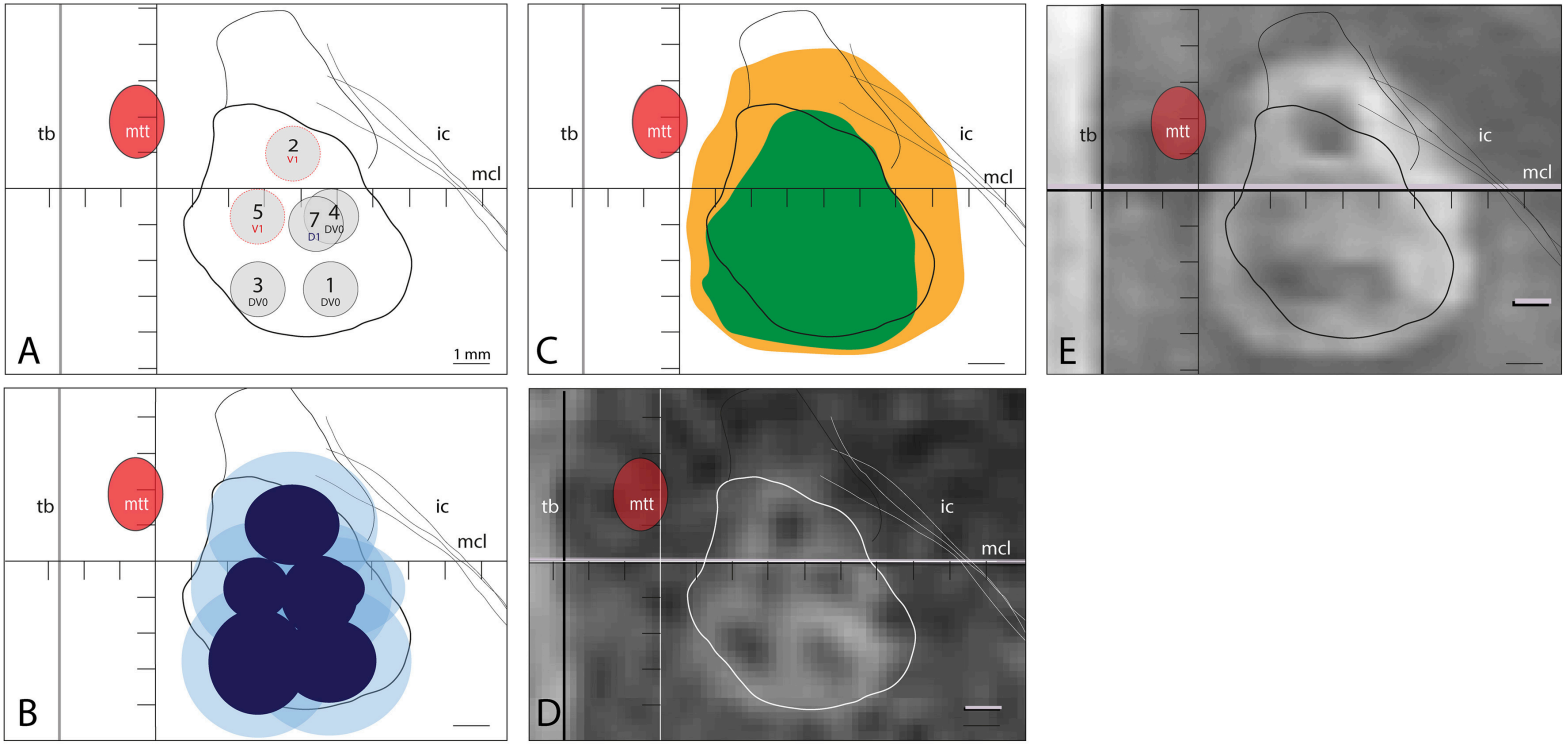
**(A)** Applied target sub-units 1,3 and 4 at plan DV0, 2 and 5 at V1 and 7 at D1. **(B)** 240 CEM thermal dose surfaces reached (dark blue) in sub-target 1 (2.6 × 2.3 mm), 2 (2.6 × 2.2), 3 (2.7 × 2.9), 4 (4.1 × 2.8), 5 (1.8 × 1.7) and 7 (2.1 × 2.2). 18 CEM thermal dose surfaces (light blue) reached in sub-target 1 (4.7 × 4), 2 (4.5 × 4.1), 3 (4.2 × 4.2), 4 (4.1 × 2.8), 5 (3.6 × 3.6), 7 (1.8 × 1.2 and 4 × 3.9). **(C)** shows the projection of the axial T2 MR lesions measured intraoperatively (green, corresponding to **D**) and at 48 h post-treatment (orange, corresponding to **E**), displayed at high magnification.

**Figure 2:**
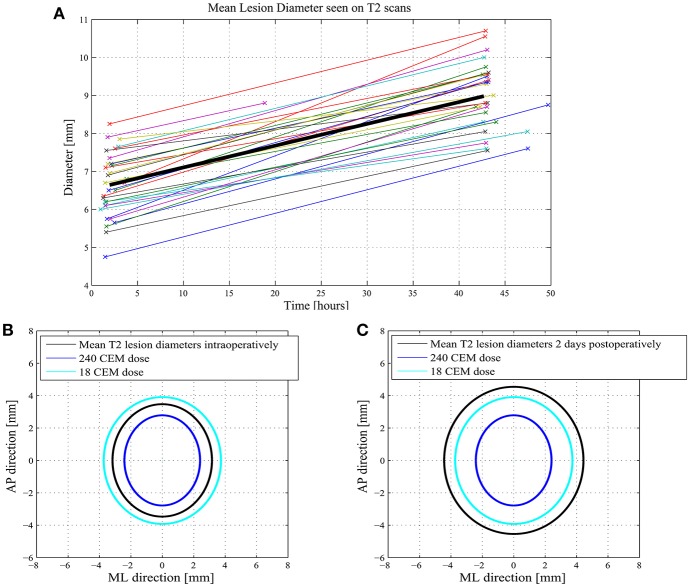
**(A)** shows 31 cases with measurements of their mean lesion diameter in axial T2 intra- and postoperatively. **(B)** shows intraoperative mean axial T2 measurements of the lesions as well as their 240 and 18 CEM thermal dose mediolateral and anteroposterior diameters. **(C)** shows the postoperative T2 lesion diameters at 2 days with their thermal doses.

Figure 1 illustrates a practical case of PTT target with 6 realized sub-targets, their 240 and 18 CEM corresponding thermal dose surfaces and their MR T2 axial images performed intraoperatively and at 48 h after the procedure. In Figure 2, we show intra- and postoperative lesion measurements in 31 cases of PTT.

Mean time between first sonication and MR T2 axial images used for intraoperative measurement was 117 ± 39.9 min and 42.7 ± 4.7 h for the postoperative scan as shown in Figure 2A for 31 consecutive cases. Mean axial T2 lesion diameter was 6.6 ± 1 mm immediately after the treatment and 9.0 ± 1.0 mm postoperatively. Mean axial T2 measurements of the lesions (*n* = 31) were 6.34 mm [SD: 1.0, CI 99% (5.88; 6.80)] in mediolateral and 6.95 mm [SD: 0.9, CI 99% (6.5; 7.3)] in the anteroposterior dimensions (black ellipse in Figures 2B,C). Forty eight hours after the procedure, they were 8.9 mm [SD:1.1, CI 99% (8.4; 9.4)] and 9.1 mm [SD: 0.9, CI 99% (8.7,9.5)], respectively. Mean measured intraoperative 240 CEM thermal dose diameters in the mediolateral direction were 4.8 [SD:1.0, CI99% (4.4; 5.3)] and 5.6 mm [SD: 0.9, CI 99% (4.4; 5.3)] anteroposteriorly (dark blue ellipse in Figures 2B,C). The 18 CEM thermal dose diameters were 7.5 mm [SD: 1.4, CI 99% (6.95; 8.16)] and 7.8 mm [SD: 1.0, CI 99% (7.3; 8.3)], respectively (light blue ellipse in Figures 2B,C).

**Figure 3:**
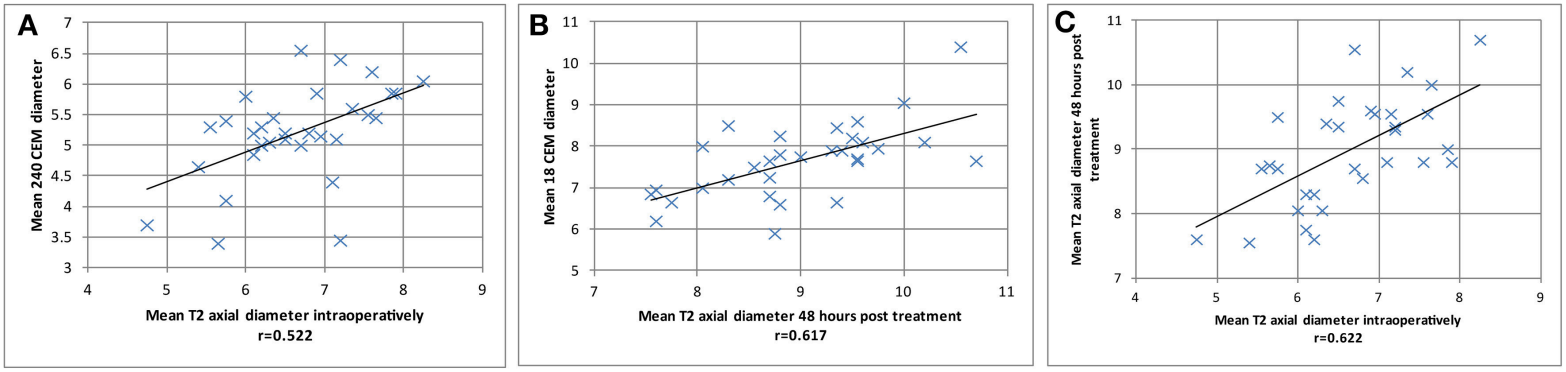
Thirty one PTT targets are displayed for correlations between mean axial T2 lesion diameter intraoperatively and the mean 240 CEM thermal dose diameter in **(A)** (*r* = 0.52). In **(B)** mean T2 axial diameter 48 h post treatment was correlated with the mean 18 CEM thermal dose diameter (*r* = 0.62). In **(C)**, mean axial T2 lesion diameter intraoperatively was correlated with mean T2 axial diameter 48 h post-treatment (*r* = 0.62).

Statistical analysis in 31 PTT targets displayed in Figure 3 revealed good correlations (1) between mean axial T2 lesion diameter intraoperatively and the mean 240 CEM thermal dose diameter (*r* = 0.52, Figure 3A), (2) between the mean axial T2 lesion diameter 48 hours post treatment and the mean 18 CEM thermal dose diameter (*r* = 0.62, Figure 3B), and (3) between the mean axial T2 lesion diameter intraoperatively and 48 h post-treatment (*r* = 0.62, Figure 3C).

**Figure 4:**
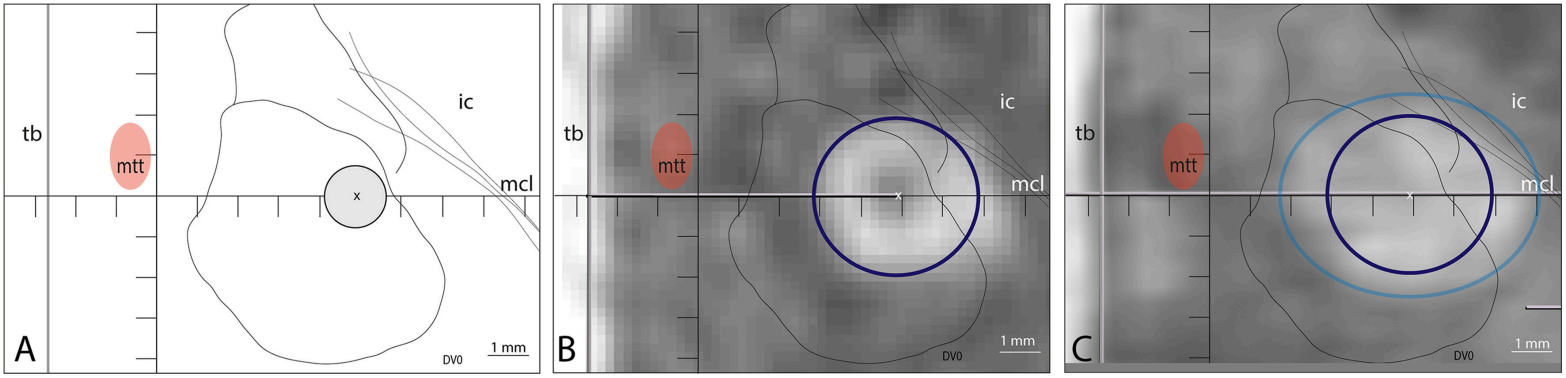
Example of insufficient target coverage of a patient treated before this study series which led to initially good but later partial therapeutic effect on the symptoms. **(A)** Target was chosen at (L7.5, MCL, DV0) and repeated sonications (*n* = 4) with temperatures ranging from 55 to 57°C and of 13 s duration were applied on the same spot. The 240 CEM thermal dose outline (dark blue circle) was projected in **(B)** on the axial T2 scan intraoperatively (4 × 3.8 mm) and both 18 CEM (light blue, 6.3 × 4.9 mm) and 240 CEM thermal dose outlines are shown in **(C)** on the T2 axial scan performed 2 days after the treatment.

**Figure 5:**
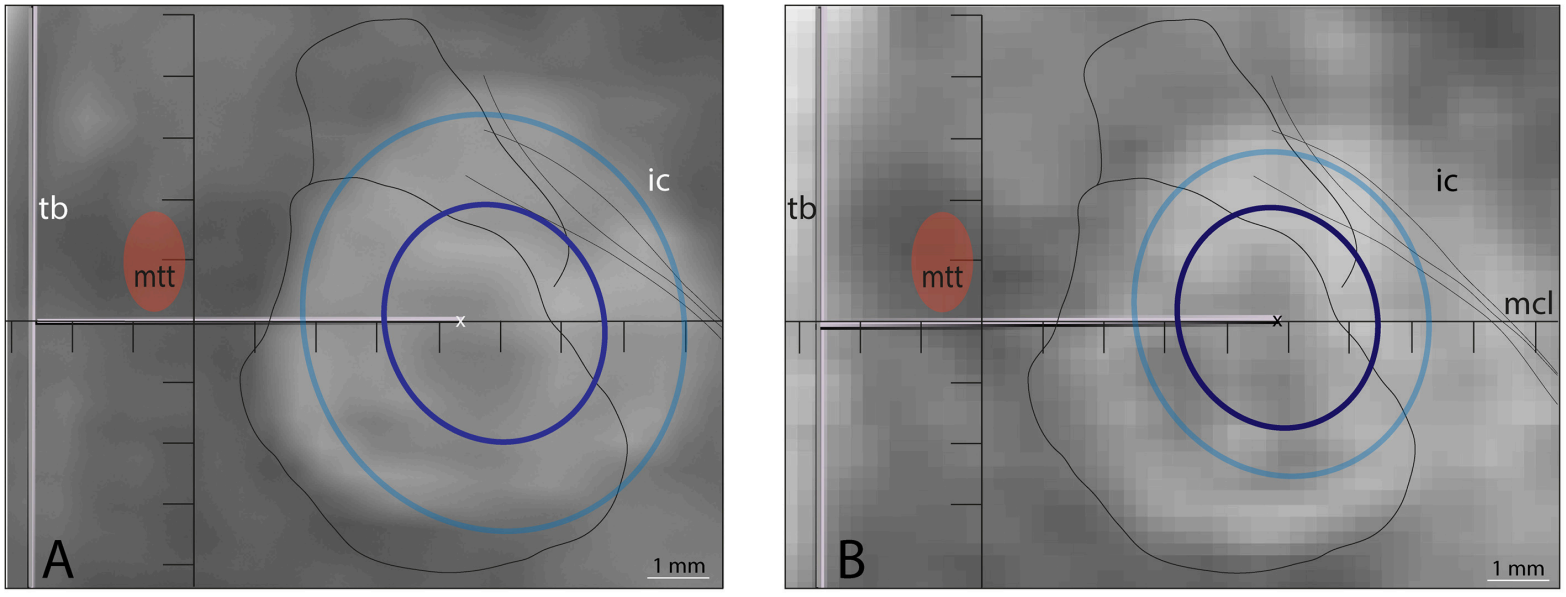
Two patients in whom longer sonication durations were applied treated before this series. Spatial coverage was increased as shown in the thermal dose values for 240 CEM but as shown in the axial T2 scan 48 h after the treatment, the hyperintense signal was difficult to associate with any thermal dose equivalent. In **(A)** temperatures reached in final sonications were 56, 56, and 54°C (sonication time: 28, 28, and 20 s, power: 450, 500, and 600 W) with final 18 CEM (light blue) and 240 CEM (dark blue) surfaces in axial projections of 6.1 × 6.8 and 3.5 × 3.9 mm, respectively. In B: temperature reached was 57°C in 24 s (450 W) for final 18 and 240 CEM thermal dose surfaces of 4.7 × 5.4 and 3.2 × 3.6 mm, respectively. In **(A,B)**, the PTT single target coordinates were L7.5, MCL, and DV0.

**Figure 6:**
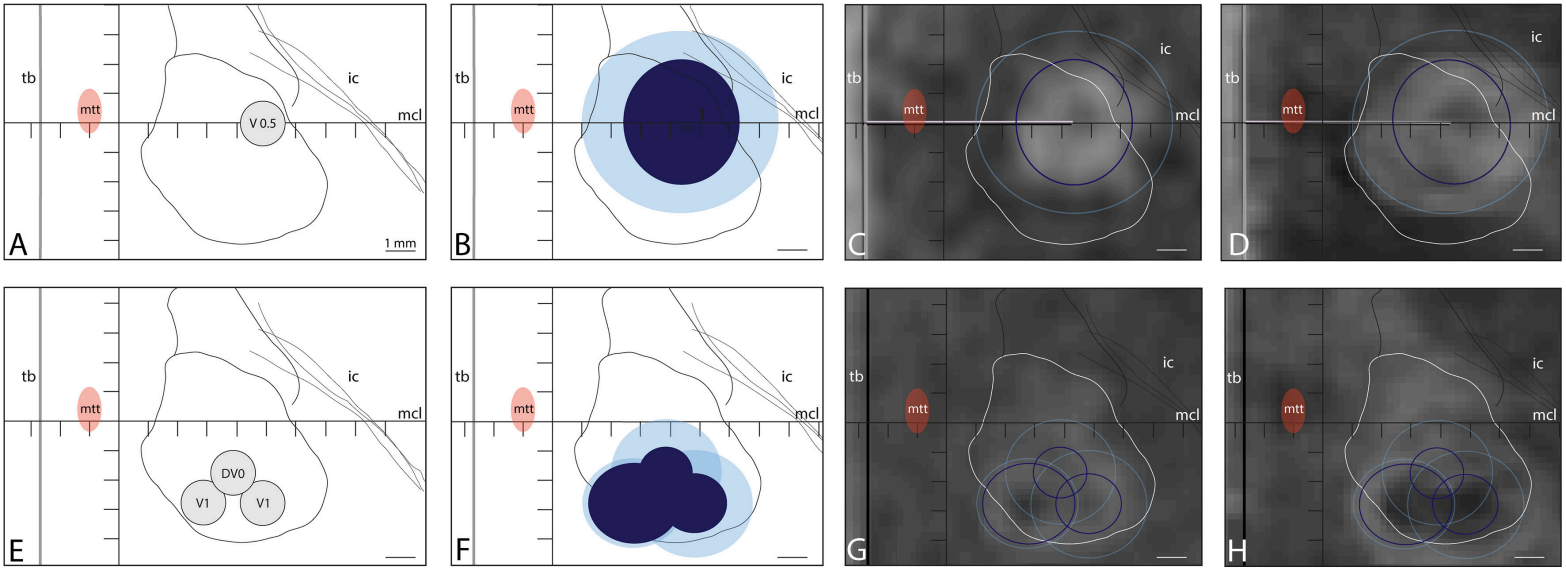
Two treatments performed in the same patient within 2 years (**A–D** and later **E–H**) because of insufficient target coverage and partial symptom recurrence. **(A,B)** show the target placed at L7.5, MCL, and V0.5. Temperatures reached in final sonications were 55, 56, 58, 57, and 55°C (sonication time: 13 s for all, power: 350, 450, and 500 W) with final 18 CEM (light blue) and 240 CEM (dark blue) thermal dose surfaces on axial projections of 6.6 × 6.1 mm and 3.9 × 4.2 mm, respectively. **(C,D)** show the lesions in axial T2 scan performed intraoperatively **(C)** and 2 days after it **(D)** with projections of their 18 and 240 CEM thermal dose outlines. In a second session, a complement of PTT was planned (E) in 3 target sub-units: 1 (L7.5, MCL-2.7, V1), 2 (L5.5, MCL-2.7, V1), and 3 (L6.5, MCL-1.7, DV0). Temperatures reached in final sonications were 55, 56, and 55°C (sonication time: 10, 10, and 16 s, power: 550, 550, and 650 W). Final 18 and 240 CEM thermal dose surfaces were shown in **(F)**. **(G,H)** show the 240 and 18 CEM thermal dose outlines projected on axial T2 scans performed intraoperatively **(G)** and 2 days after it **(H)**.

An example of insufficient target coverage despite repeated sonications in a patient treated before this study series is illustrated in Figure 4. In a second step before this series, longer sonication durations were applied. Spatial coverage was significantly increased as shown in Figure 5 but was nevertheless insufficient in some patients. Figure 6 illustrates how our current approach can be successfully applied to complement an insufficiently covered PTT target performed previously.

## Discussion

Targeted thresholds of 18 and 240 CEM are based on experimental data in the rabbit and primate brain summarized by MacDannold and co-workers (3, 4). The thermal dose threshold of 240 CEM is thought to represent the LD100, or 100% probability to achieve necrosis at the corresponding isothermal surface, and the 18 CEM value represents a 50% probability for thermal damage. Imaging and histological correlations with a thermal dose based approach have been published in animals (4, 11–17) and in humans (18, 19). Little is known about tissue specific (white matter vs. gray matter) variations in the human brain as well as tissue changes with increasing age. Hints about different tissue thresholds can already be found in the older ultrasound literature (20, 21). The approach described here concerns a fiber tract target, in which a relevant part of heat diffusion may take place along its course. We expect more homogeneous perilesional heat diffusion patterns in nuclear targets (thalamus and pallidum). These observations warrant future detailed analyses of perilesional heat diffusion in nuclear targets.

Bringing together the correlative data of this study and histological animal data (4), the following can be proposed. Intraoperative T2 imaging zones I and II (10) correspond to the sum of the 240 CEM and a part, the most central, of the 18 CEM thermal dose area (Figure 2B), and thus to the 6 to 7 mm large thermolesioned tissue domain Figure 3A shows a good correlation between the intraoperative T2 imaging and the 240 CEM thermal dose area. The comparison between the T2 zone I and II surface areas intraoperatively and at 2 days allows to document the amount of edema arising during the first 2 postoperative days (Figures 2C, 3C). Figure 3B shows a good correlation between the T2 imaging of zone I and II area after 2 days and the 18 CEM thermal dose area. These areas are larger than the thermolesioned domain, and they correlate well with each other. This allows to foresee at the end of the intervention how large the zone I and II will be at 2 days, and to know that their extension is non-lesional and due to the development of intralesional cytotoxic and perilesional vasogenic edema. A mentioned above, it is only the most central part of the 18 CEM area which contains thermolesioned tissue. Concerning study limitations, histological data which have allowed to develop the thermal dose based lesioning parameters are all derived from animal experiments, and should be transposed with care to humans, as they surely cannot include for example the changes underwent by the aging human brain tissue. Secondly, there are no volume calculations because all measurements were performed only on axial planes, i.e., two-dimensionally. Thirdly, we still lack knowledge about the cumulative effect of thermal doses: there is rising but not yet quantified evidence that they can be summed up over one single target, thus allowing a successful thermal lesioning even in patients with unfavorable skull absorption preventing the application of a 240 CEM thermal dose in a single application.

## Conclusion

This study presents our current approach to the MRgFUS pallidothalamic lesioning which was developed because the two strategies of application repetitions and increase of application duration failed to prevent symptom recurrences or partial symptom control. It takes into account interindividual variability, and is characterized by the applications of multiple small thermal lesions using shortest possible durations, and distributed in a preplanned manner to optimize target coverage. Using thermal dose monitoring rather than energy- or temperature-based approaches sticks with the biophysics of focused ultrasound based tissue thermal lesioning and provides a correlative and predictive dimension. This one is essential when considering sufficient lesion coverage of targets and safety for surrounding structures in MRgFUS functional neurosurgical interventions.

Our clinical results (in preparation) show as expected a clear-cut reduction of symptom recurrences or partial symptom control over the first postoperative year.

## Ethics Statement

No ethical approval was sought because MRgFUS PTT is approved by the Swiss Health State Department and covered by swiss social insurances. All patients treated with this protocol signed an informed consent form after having been fully informed about the treatment, its results and risks.

## Author Contributions

MG, DJ, and DM: conception and design. MG and DM: acquisition of data. MG, DM, CF, and DJ: analysis and interpretation of data. All authors: drafting the article, critically revising the article, and reviewed submitted version of manuscript. MG: approved the final version of the manuscript on behalf of all authors. MG and CF: statistical analysis. DM: administrative/technical/material support.

### Conflict of Interest Statement

MG, DM, and DJ were employed by company SoniModul Ltd, Center of Ultrasound Functional Neurosurgery, Solothurn, Switzerland. The remaining author declares that the research was conducted in the absence of any commercial or financial relationships that could be construed as a potential conflict of interest.

## Abbreviations

ICL: intercommissural line
MTT: mammillothalamic tract
IC: internal capsule
CEM: cumulative equivalent min at 43°C
MCL: mid-commissural line
MRgFUS: MR-guided high intensity focused ultrasound
PTT: pallidothalamic tractotomy
PD: Parkinson's disease.
